# Evaluation of sputum eosinophil count as a predictor of treatment response to mepolizumab

**DOI:** 10.1183/23120541.00560-2021

**Published:** 2022-05-03

**Authors:** Ian D. Pavord, Roland Buhl, Monica Kraft, Charlene M. Prazma, Robert G. Price, Peter H. Howarth, Steven W. Yancey

**Affiliations:** 1 Respiratory Medicine Unit and Oxford Respiratory National Institute for Health Research Biomedical Research Centre, Nuffield Dept of Medicine, Oxford, UK; 2 Pulmonary Dept, Johannes Gutenberg University of Mainz, Mainz, Germany; 3 Dept of Medicine, University of Arizona, Tucson, AZ, USA; 4 Global Respiratory Franchise, GSK, Research Triangle Park, NC, USA; 5 Biostatistics, GSK, Stevenage, Hertfordshire, UK; 6 Respiratory Medical Franchise, GSK, Brentford, UK; 7 Respiratory Therapeutic Area, GSK, Research Triangle Park, NC, USA

## Abstract

For patients with asthma, eosinophilic airway inflammation is associated with poor lung function, increased disease severity, reduced quality of life and increased risk of exacerbations [1, 2]. As such, several biologic therapies targeting cytokines involved in eosinophil survival and activation have been developed, with the aim of reducing eosinophilic inflammation [3]. The response to these cytokine-targeting biologics has typically been assessed by monitoring clinical outcomes. Reductions in blood eosinophils have also been monitored [2], since these cells are easily accessible and are reflective of eosinophilic airway inflammation [4, 5]. However, the utility of sputum eosinophils as a biomarker for assessing the therapeutic response to biologic therapies remains an area of ongoing scientific debate and has been largely unexplored, owing to logistical challenges associated with their collection and measurement.

## Abstract 2

**In patients with severe eosinophilic asthma and sputum eosinophil counts of ≥3–<30%, sputum eosinophils may not represent a more useful biomarker than blood eosinophils for predicting clinical treatment response to mepolizumab** https://bit.ly/3pOTw93

*To the Editor:*

For patients with asthma, eosinophilic airway inflammation is associated with poor lung function, increased disease severity, reduced quality of life and increased risk of exacerbations [1, 2]. As such, several biologic therapies targeting cytokines involved in eosinophil survival and activation have been developed, with the aim of reducing eosinophilic inflammation [3]. The response to these cytokine-targeting biologics has typically been assessed by monitoring clinical outcomes. Reductions in blood eosinophils have also been monitored [2], since these cells are easily accessible and are reflective of eosinophilic airway inflammation [4, 5]. However, the utility of sputum eosinophils as a biomarker for assessing the therapeutic response to biologic therapies remains an area of ongoing scientific debate and has been largely unexplored, owing to logistical challenges associated with their collection and measurement.

Mepolizumab is an anti-interleukin-5 monoclonal antibody approved as an add-on treatment to standard of care for patients with severe eosinophilic asthma [6]. Randomised controlled trials in this patient population have demonstrated that compared with placebo, mepolizumab reduces exacerbation frequency, and improves lung function, asthma control and health-related quality of life [7–10]. In a phase IIa, multicentre, randomised, open-label, parallel-group, repeat-dose study conducted in patients with blood eosinophil counts >300 cells per μL (GlaxoSmithKline (GSK) identifier MEA114092; www.clinicaltrials.gov identifier NCT01366521), mepolizumab reduced blood and sputum eosinophils in a dose-dependent manner after 3 and 7 days of treatment, respectively [8]. In the subsequent phase IIb/III, multicentre, randomised, double-blind, placebo-controlled DREAM study (GSK identifier MEA112997; www.clinicaltrials.gov identifier NCT01000506), dose-dependent reductions in blood and sputum eosinophils were observed across a 10-fold intravenous (*i.v.*) mepolizumab dose range [9]. However, the relative reduction from placebo in exacerbation rate was similar across the full mepolizumab dose range tested. We therefore sought to further investigate the relationship between blood and sputum eosinophil counts and clinically meaningful treatment responses in patients receiving mepolizumab *i.v.*

This *post hoc* analysis included data from patients with severe eosinophilic asthma who provided sputum samples during the DREAM study; details of the study design have been reported previously [9]. Briefly, patients (N=616) were randomised (1:1:1:1) to receive placebo or 75, 250 or 750 mg mepolizumab *i.v.* in addition to standard of care, every 4 weeks for 52 weeks. Eligible patients were ≥12 years of age with severe eosinophilic asthma (confirmed by a clinical diagnosis of asthma plus evidence of eosinophilic inflammation) and two or more exacerbations in the year preceding enrolment despite receiving standard of care. A subset of patients (n=94) provided sputum samples at baseline, weeks 4, 16 and 52, and at follow-up. Sputum samples were processed within 2 h of collection and sputum eosinophil counts were measured at a centralised site. Within this subset of patients providing sputum samples, annual rates of clinically significant exacerbations were compared between treatments for patients stratified by thresholds of baseline sputum and blood eosinophil counts (as continuous variables) using a negative binomial model with covariates of treatment group, geographical region, exacerbations (as an ordinal variable), maintenance oral corticosteroid use (yes or no), baseline percent predicted forced expiratory volume in 1 s, baseline eosinophils, baseline eosinophils by treatment interaction and logarithm of time on treatment as an offset variable; a pre-specified log transformation was applied to sputum and blood eosinophil counts before analysis.

Of the 616 patients in the intent-to-treat (ITT) population of DREAM with baseline blood eosinophil count data available, 86 patients with baseline sputum eosinophil samples (mepolizumab: n=62; placebo: n=24) and 94 patients with baseline blood eosinophil samples (mepolizumab: n=67; placebo: n=27) were included in the analyses. All doses of mepolizumab *i.v.* were pooled for the analyses. The majority (66 out of 86, 77%) of patients with baseline sputum samples had sputum eosinophil counts ≥3%; those patients with higher baseline sputum eosinophil counts (≥3% *versus* <3%) also had higher geometric mean blood eosinophil counts (320 *versus* 120 cells per μL) and immunoglobulin E concentrations (196 *versus* 77 IU·mL^−1^) at baseline. Patient characteristics such as age, sex and body mass index were generally similar across treatment arms and irrespective of baseline sputum or blood eosinophil count.

Following 52 weeks of treatment, contrary to previously published data [11], results of the modelling analysis predicted a decrease in exacerbation rates among the placebo group with increasing baseline sputum eosinophil count (3.13, 2.84, 2.48 and 2.00 events per year at counts of 3%, 5%, 10% and 30%) (figure 1a). This result may be due to the small number of patients with baseline sputum eosinophil data available or may present an interesting new finding for further investigation. Conversely, predicted exacerbation rates increased in the placebo group with increasing baseline blood eosinophil count (2.56, 3.00, 3.36 and 3.69 events per year at counts of 150, 300, 500 and 750 cells per μL) (figure 1b). For patients receiving mepolizumab, predicted exacerbation rates decreased with increasing baseline sputum and blood eosinophil count (0.85, 0.80, 0.74 and 0.65 events per year at sputum eosinophil counts of 3%, 5%, 10% and 30%; 0.97, 0.86, 0.79 and 0.73 events per year at blood eosinophil counts of 150, 300, 500 and 750 cells per μL) (figure 1b).

**FIGURE 1:**
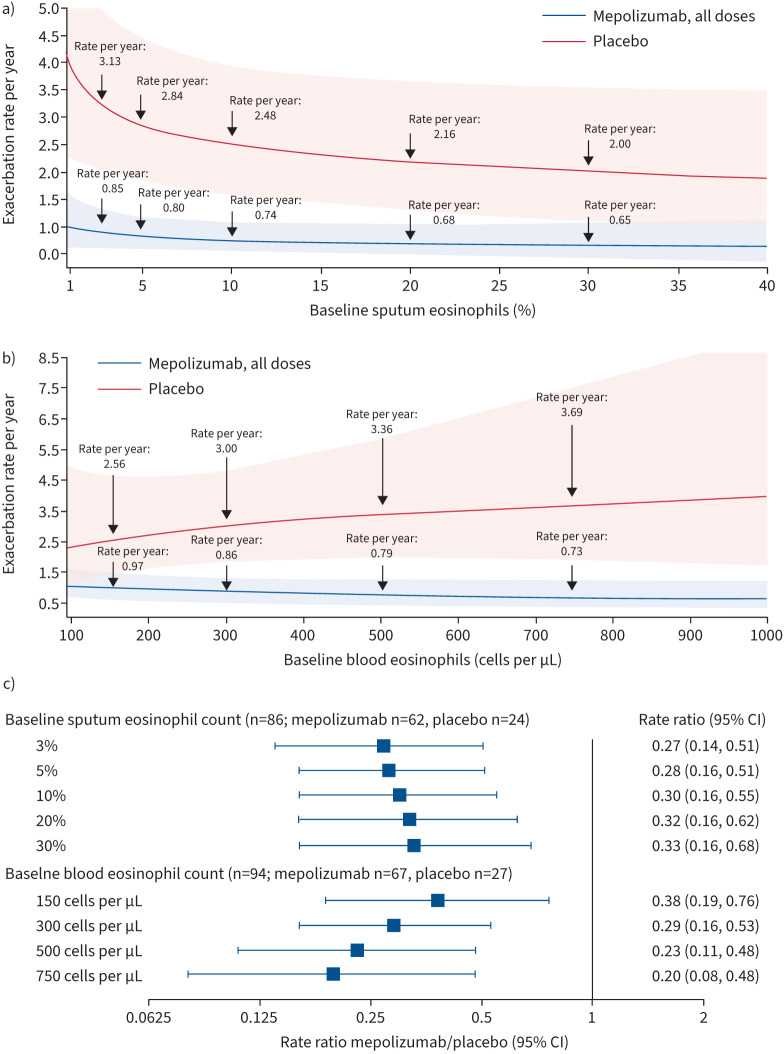
Predicted annualised rates of clinically significant exacerbations at week 52, by a) baseline sputum eosinophil count and b) baseline blood eosinophil count; c) rate ratio (mepolizumab/placebo) of clinically significant exacerbations, by sputum and blood eosinophil count thresholds. Shaded areas in panels a) and b) represent 95% confidence intervals for predicted rates; arrows represent predicted events per year at baseline eosinophil thresholds. The analyses of clinically significant exacerbations included all patients with sputum samples and a) sputum eosinophil data available at baseline (mepolizumab: n=62 (n=18, 23 and 21 in the 75, 250 and 750 mg intravenous (*i.v.*) arms, respectively); placebo: n=24) and b) blood eosinophil data available at baseline (mepolizumab: n=67 (n=20, 24 and 23 in the 75, 250 and 750 mg *i.v.* arms, respectively); placebo: n=27). Owing to small sample sizes, statistical testing was not performed on these data.

With regards to clinical benefit, exacerbation rates were reduced by 62–80% with mepolizumab *versus* placebo across the range of baseline sputum and blood eosinophil thresholds (figure 1c). Lower baseline sputum eosinophil counts had little impact on reductions in exacerbation rate with mepolizumab *versus* placebo, compared with higher baseline sputum eosinophil counts. By contrast, reductions in exacerbation rate with mepolizumab *versus* placebo appeared to increase with increasing baseline blood eosinophil count (figure 1c); a similar trend was seen in the DREAM ITT population (data not shown).

A previous study has indicated that maintaining sputum eosinophils <3% can reduce the frequency of asthma exacerbations [11]. In our analysis, reductions in exacerbations were seen with mepolizumab *versus* placebo irrespective of baseline sputum eosinophil count. In contrast, exacerbation reductions with mepolizumab *versus* placebo were largest among patients with higher baseline blood eosinophil counts, consistent with previous analyses of mepolizumab and other biologics in the treatment of eosinophilic asthma [12–15].

The main limitations of this analysis were the small number of patients with available sputum data and the resulting data variability; this analysis may be insufficiently powered to assess the impact of sputum eosinophilia on treatment response at the higher or lower limits of <3% (n=12 mepolizumab, n=8 placebo) or ≥30% (n=18 mepolizumab, n=9 placebo), owing to the small number of patients with baseline sputum eosinophil counts in these ranges. Moreover, patients receiving all doses of mepolizumab (75, 250 or 750 mg *i.v.*) in DREAM were pooled for this analysis, owing to the small number of patients with available sputum data in each treatment arm. As a result, some patients receiving mepolizumab doses higher than the currently approved dose for severe eosinophilic asthma (100 mg subcutaneously) were included in this analysis. Further studies will therefore be required to definitively investigate our findings in a larger number of patients. The *post hoc* nature of this subgroup analysis must also be taken into consideration when interpreting results. Nevertheless, our findings demonstrate that although patients with severe asthma and sputum eosinophilia are likely to experience clinically meaningful reductions in exacerbations with mepolizumab, these reductions are seen irrespective of baseline sputum eosinophil count. Given the aforementioned limitations, these data suggest that particularly in those patients with sputum eosinophil counts of ≥3– <30%, sputum eosinophils may not represent a more useful biomarker than blood eosinophils for predicting clinical treatment response to mepolizumab in patients with severe eosinophilic asthma.
